# Heat Shock Proteins in the Human Eye

**DOI:** 10.1155/2010/479571

**Published:** 2011-03-02

**Authors:** Lærke Urbak, Henrik Vorum

**Affiliations:** Department of Ophthalmology, Aalborg Hospital, Aarhus University Hospital, Hobrovej 18-22, 9100 Aalborg, Denmark

## Abstract

Heat shock proteins (Hsps) are believed to primarily protect and maintain cell viability under stressful conditions such as those occurring during thermal and oxidative challenges chiefly by refolding and stabilizing proteins. Hsps are found throughout the various tissues of the eye where they are thought to confer protection from disease states such as cataract, glaucoma, and cancer. This minireview summarizes the placement, properties, and roles of Hsps in the eye and aims to provide a better comprehension of their function and involvement in ocular disease pathogenesis.

## 1. Introduction

In order to maintain the health of a cell, it is crucial to preserve the integrity of its proteins. The differing ocular cell types have specific mechanisms that facilitate proper folding of proteins, refolding of partly folded or misfolded proteins, and the disposal of irreversibly damaged proteins. Indeed, the heat shock proteins (Hsps), which act as molecular chaperones are extremely important contributors to these crucial cellular protein processes [1]. These molecular chaperones, which are expressed under normal conditions in the eye may be significantly upregulated with elevated temperatures or other stresses [2]. 

Hsps are primarily divided into groups according to their molecular mass prior to further subdivision. For example, Groenen et al. [3] classify Hsps into four groups: small Hsps, Hsp60, Hsp70, and Hsp90 where the numbers refer to the molecular mass in kilodaltons. Others have divided Hsps into seven groups: small Hsps, Hsp40, Hsp60, Hsp70, Hsp90, Hsp100, and large Hsps [4, 5]. 

In response to an ocular stress or injury, Hsp expression in the eye may be increased in order to possibly provide a cytoprotective action by facilitating the refolding of damaged proteins for instance [6]. This response, which can occur acutely or chronically, has been shown to be induced by a number of ways that include elevated temperatures [5], ischemia [7], osmotic stress [8], and oxidative stress [9]. 

**Figure 1:**
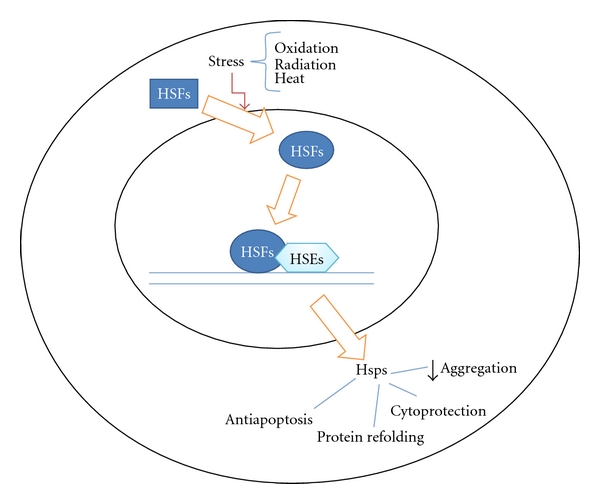
Regulation of stress induced Heat Shock Proteins (Hsps) by Heat Shock Factors (HSFs) and Heat Shock Elements (HSEs). The outer circle defines the cell membrane, and the inner circle defines the nucleus.

The ocular heat shock response is activated by Heat Shock Factors (HSFs) (Figure 1) that are divided into groups of which HSF1 is the best studied. When stress is induced in the cells of the eye, HSF relocalizes to the nucleus, forming a granule-like structure [10] and binds with high affinity to Heat Shock Elements (HSEs) that are placed in the promoter region of the target gene, which promotes transcription of Hsps [11]. Thereby, the regulation of the ocular heat shock response is essentially regulated at the transcriptional level by the activities of HSF. In addition, HSF1 has been shown to be negatively regulated by feedback control through interactions with the Hsps themselves [10]. 

Beside reducing the aggregation and degeneration of ocular cellular protein, one of the key functions of Hsps is to prevent cell apoptosis, which they achieve in different ways. For example, Hsp70 and its cochaperone Hsp40 prevent translocation of Bax to the mitochondria during NO-mediated apoptosis [12]. Both Hsp70 and Hsp90 can halt the progression of apoptotic signaling by negative regulation. They directly interact with Apaf-1 apoptosome and thereby prevent its oligomerization and/or association with procaspase-9 [13]. 

It has been shown that a decrease in Hsps by interference at the RNA level leads to a decrease in the longevity of the cells [14]. This may have implications with regard to the prevention of aging diseases of the eye. However, it has also been shown that tumor cells express a higher level of Hsps compared to nontumor cells which may suggest that Hsp expression is involved in tumor formation [15] though this requires further clarification. 

In this review, we will focus on Hsps function in the normal human eye and their involvement in ocular disease. 

## 2. Heat Shock Proteins in the Normal Eye

### 2.1. Cornea

The main functions of the Hsps in the cornea are unclear. In a recent study upon sixteen human corneas, Gain et al. [16] found Hsp27, Hsp60, Hsp70, and Hsp90 expressed in, respectively, 97%, 59%, 88%, and 85% of samples. Under normal conditions, Hsp70 functions as an ATP-dependant molecular chaperone [17]. When the eye is under stress the synthesis of Hsp70 increases to cope with the increased amount of unfolded or denatured proteins [18]. This phenomenon is also observed in the cornea of humans [4], where Hsp70 as part of the stress response can inhibit apoptosis or DNA damage and thereby increases cell survival [4]. Ishihara et al. [19] found that following laser treatment, Hsp72 showed up in the regenerative cornea epithelium of the rabbit, though the basis of this finding was not clear. 

It has been observed that under normal conditions in the corneal epithelium, Hsp27 is mainly present in a nonphosphorylated form [20]. However, when the cornea is stressed with UVB irradiation, Hsp27 phosphorylates and translocates [20], which may serve to protect or repair the corneal epithelial cells [20, 21]. 

Hsp47 has also been observed in the endoplasmic reticulum and small vesicles of the human cornea [22]. In normal conditions, Hsp47 functions as a type 1 procollagen chain selector or folder, since and may be involved in its synthesis [23], just as it is part of the translation-translocation of the type 1 procollagens in the body [24]. Under stressful conditions, Hsp47 has been suggested to aid the control of procollagen in the eye [25] and is observed to be present when corneal structure damage has occurred [26]. 

As with the lens, it is essential for the cornea to remain stable and transparent. The main structural proteins of the lens are the crystallins; some of which also have heat shock protein function. However, the cornea also contains another crystallin type [27], which so far has not been shown to have heat shock ability (Table 1). 

### 2.2. Lens

Hsp40, Hsp70, and the two types of small Hsps (sHsps) have been so far localised to the lens. Hsp27 and alpha-crystallin have been shown to be present in human fetal as well as older persons lenses [28] (Table 1). Beta-B2 crystallin is also a significant component of the lens [29]. 

Bagchi et al. [28] found Hsp70 to be present in the nonstressed lens. Rothstein and Bagchi [30] found Hsp70 to be mainly present in the epithelial and superficial cortical lens fibers and together with hsp40, are found in areas with significant protein synthesis. Hsp40 is also found in the epithelial and superficial cortical fibers of the lens [28]. Hsp40 and -70 have been found to decline with age [28], which can lead to structural changes in the lens that might play a part in the development of diseases indirectly. Hsp27 was found in most of regions of the lens and did not decline with age [28]. 

Alpha-crystallin is a sHsp that is a major protein component of the vertebrate lens [31]. The lens fiber cells and their proteins have little regenerative possibilities [32]. In the endeavor to maintain the transparency of the lens, the lenticular cells must survive and preserve their structural and functional integrity over a lifetime. When proteins denature, they aggregate and form large insoluble particles. Thus, denaturation of protein in the lens, as a result of an oxidative insult from ultraviolet radiation for instance, will obviously be detrimental to the transparency of the lens [32]. Maiti et al. [33] have shown that alpha-crystallin is stable up to 100°C. However, Heys et al. [34] suggest that absorption of solar radiation in a warm climate over a lifetime leads to heat induced crystalline denaturation. 

Like other proteins alpha-crystallin has an N- and C-terminal. The C-terminal includes the alpha-crystallin domain, which is equal to all sHsps [35], and the N-terminal has been shown to be easily modified [3]. Laganowsky et al. [36] demonstrated that the alpha-crystallin has a flexible structure, which is an advantage when having to interact and bind different molecules. Raman et al. [37] have demonstrated that with increased temperature alpha-crystallin was structurally reorganized to have a hydrophobic surface that can bind the nonnative protein and thereby prevent aggregation. 

Unlike most other Hsps, sHsps are ATP independent [38] and function as a holding chaperone by binding to unfolded proteins under stress [39] and stabilizing the protein in a standby position without refolding it [40, 41]. The refolding is performed later by the larger Hsps [2]. It is not clear whether the alpha-crystallin in the lens works in the same way as the other sHsp or has a refolding function itself, but it is proven that alpha-crystallin prevents aggregation and the proteins are refolded similar to their native state [32]. 

Alpha-crystallin is an example of gene sharing within the same tissue and the same cell. In the lens where it is expressed at very high levels, alpha-crystallin serves as a refractive element and as a protective Hsp by preserving the stability and transparency of the lens [32]. 

### 2.3. Retina

Hsp70 is found in human fetal retina in week 20–33 of development, which is the same period when the nuclear layers are formed. This could suggest that Hsp70 plays an important role in normal retinal development [42], which is the same conclusion made from a study performed on rat retina by Kojima et al. [43]. Additionally, another rat study suggested that Hsp70 is necessary to protect retinal cells against bright light [44]. 

Tezel and Wax [45] incubated hsp27 antibody with a human retina. This caused cell death suggesting that the hsp27 antibodies inhibited Hsp27 antiapoptotic function through binding. This also supports the finding of Hsp27 in normal retina [7]. Tezel and Wax [45] similarly observed that the major loss of retinal neurons by Hsp27 antibody-mediated apoptosis occurred in the ganglion cell layer, suggesting that Hsp27 plays an important role here. 

Though there have not been many Hsp studies performed upon human retinal tissue, many animal studies have demonstrated a variety of Hsps in the retina. Tanaka et al. [46] studied the eyes of rat embryos. In the retina of the rat embryo, heat shock cognate protein (HSC) 70, Hsp84, and Hsp86 mRNAs were found. When observed at a high magnification it was noted that all three Hsps were observed in the neuroblastic layer of the retina. Furthermore, Hsp84 was also observed in the retinal pigment epithelium. However, only HSC70 and Hsp86 were also found in the mature retina of rats. Others have also found Hsp70 in animal studies [47–49] just as Hsp40 [47] and Hsp90 [48] have been previously observed (Table 1). 

### 2.4. Choroid

Zamora et al. [50] found Hsp-beta1 in the choroidal endothelial cells of the human eyes during a comparative proteomic study of the retina and choroid (Table 1). 

## 3. Heat Shock Proteins Involved in the Pathogenesis of Ocular Disease

### 3.1. Cornea

One of the corneas most important function is to maintain its transparency. Scarring from wounds in the corneal epithelium can decrease the transparency of the cornea and thereby reduce vision. When damaged, the remaining corneal epithelium migrates to the wounded area in the attempt to repair the defect. Ko et al. [51] suggested that Hsp70 plays a role in the wound healing process of the human cornea. They demonstrated that a fibronectin derivative phosphorylated and upregulated the amount of Hsp70 in human corneal epithelial cells and Hsp70 seemed to be lacking in wounds that heal slowly [51]. Hsp70 has also been demonstrated to act as a significant factor in wound healing of the skin [52] and in corneas of animals [53, 54] (Table 1). 

UVB radiation is well known to have the ability to damage DNA bonds and cause cellular injury [55, 56].The cornea, as one of the most outermost tissues, absorbs most of the radiation in the UVB range, which gives protection to the internal structures [57]. Both acute and chronic exposure to UV radiation can result in eye diseases [58, 59]. Since UV radiation mediates the type of stress that Hsps normally respond to, it was postulated that Hsps play an important role in protecting the cornea itself against UV radiation. Indeed, following exposure to UVB radiation, it has been shown that the nonphosphorylated Hsp27 isoform rapidly undergoes phosphorylation and translocation. This response may be an attempt by Hsp27 to protect the UVB-exposed corneal cells from apoptosis [20]. Furthermore, in another study where the corneal epithelial cells were preconditioned with arsenite, an Hsp27 promoter, prior to UVB exposure, the UVB-induced cell death and DNA damage were reduced [21].

### 3.2. Lens

Many different causes of cataract have been found. In the hereditary form, different mutations can cause cataract [60]. One such mutation can occur in genes that encode alphaA- and alphaB-crystallin, which might decrease alpha-crystallins ability to prevent protein aggregation and thereby cause a reduction in the transparency of the lens [61, 62]. 

Alpha-crystallin is known to bind to denatured proteins in the lens, and this function may be impaired over time [63]. Heys et al. [34] showed that alpha-crystallin becomes insoluble by about the age of 40. In the perspective that the alpha-crystallin changes to a hydrophobic surface structure with increased temperature [37], it could be postulated that this protein loses its molecular chaperone abilities with ageing. Lack of this function leads to protein aggregation, which again leads to decreased transparency of the lens and eventual cataract. Additionally, the structural change of alpha-crystallin might also play a role in the development of presbyopia [34]. 

In a study of patients with atopic and nonatopic cataract, there was found to be an increased amount of stress-response protein (Hrp) 60 in those with atopic cataract [64]. Previously, it had been observed that Hrp60 is located in the mitochondrial membrane [65] and since a lens with atopic cataract had an increased amount of mitochondrias [66], it could be suggested that the increased energy produced by the mitochondria provoked an increased Hrp60 to protect the cells (Table 1).

### 3.3. Retina

Age-related macular degeneration is the main cause of vision loss in the elderly. Oxidative damage leads to protein damage, aggregation, and even death of the cells in the retina and choroid [67]. Strunnikova et al. [68] hypothesized that Hsp27 plays a role in age-related macular degeneration (AMD) after they found high levels of Hsp27 in the ganglion cells, retinal pigment epithelium, and photoreceptors following injury. Increased amounts of alpha-crystallin have also been found in AMD compared to age-matched control tissue [69] suggesting that the molecular chaperone function may be important in the pathogenesis of this common disease. However, the alpha-crystallin was found extracellular where as the molecular chaperone function normally takes place intracellular. This could suggest that alpha-crystallin is involved with the damage or denatured proteins in Bruch's membrane or that it could be originating from an intracellular source [69] (Table 1). 

### 3.4. Ocular Tumors

Alpha-crystallin has been localized to retinoblastoma cells in which Pineda et al. [70] discovered alpha B-crystallin to be in high expression. However, Kase et al. [71] only found this subgroup in a small amount, with alpha A-crystallin expressed in the cytoplasm of tumor cells. Kase et al. also showed that the apoptosis was less in cells with high alphaA-crystallin. Jiang et al. [72] found Hsp70 and -90 in retinoblastoma cells. Chen et al. [73] suggested that Hsp75 plays a role in retinoblastoma development since it can refold denatured retinoblastoma protein to its native form and the two are found in a complex when the cell undergoes mitosis and is exposed to elevated temperatures. 

An increased amount of Hsp beta1, -60, -70, and -90 have been found in uveal melanoma material from one patient [74]. Zuidervaart et al. [75] found Hsp27 elevated in a study with material from one patient, and Missotten et al. [76] found the same in a study with 38 patients material, while Coupland et al. [77] found a lower or negative amount of Hsp27 in seven uveal melanomas with monosomy 3. None of the studies found a connection between Hsp27 expression and the prognostic parameters. 

Just as for retinoblastoma and uveal melanoma, Hsps have been found to be overexpressed in various other cancers in comparison to normal tissue suggesting that the increased chaperone expression contributes to oncogenesis at several levels [78]. One of the Hsps main known functions is preventing apoptosis, which can also lead to the undesirable survival of mutated cells. But, it has also been proven that different Hsps play a role in other procancer mechanisms such as generating growth signals, increasing the number of cell divisions, promotion of tumor invasive capacity, and angiogenesis via stabilizing the transcription factor H1F1*α* and vascular epithelial growth factor [79]. 

However, a thorough knowledge of Hsp participation in tumors is still lacking. Overexpression of Hsps in tumors could occur as an attempt to optimize the environment, or it could be a result of the molecular chaperone function to maintain the normal cell environment. More studies need to be undertaken on how Hsp influence and regulate cancer development [80] (Table 1). 

### 3.5. Glaucoma

Chronic ischemia of the retina secondary to reduced vascular perfusion is recognized to be a cause of glaucoma pathogenesis, particularly in the form associated with normal intraocular pressure [81]. Apoptosis is a mechanism of retinal cell death in glaucomatous optic neuropathy [82, 83]. Immunohistochemical analysis demonstrated that Hsp60 and -27 were greater in glaucomatous eyes than in nonglaucomatous eyes of humans [84] suggesting that Hsp60 and -27 may be part of a defense mechanism that is activated in glaucomatous optic neuropathy. After inducing an ischemic period by acutely increasing the intraocular pressure, an elevated amount of Hsp27 and -72 have been demonstrated in the retinal ganglion cells of rats [85]. Similarly, an increase in Hsp27 and phosphorylated Hsp27 were observed mainly in glia cells after increased intraocular pressure in rats and mice [86]. It has also been shown that inducing Hsp72 in a rat model of acute glaucoma correlates with increased survival of retinal ganglion cells [87]. However, it has also been demonstrated in ex vivo and in vitro retinal models that glaucoma is associated with elevated serum antibodies to sHsps, and these antibodies facilitate apoptosis of retinal cells [88]. According to Tezel and Wax [45], the Hsps may activate the T cells that facilitate retinal ganglion cell apoptosis. The enhanced expression of Hsp in glaucomatous eyes may consequently submit them as immune targets that cause neuronal apoptosis and thereby diminish the protection that Hsps confer [45, 89].

## 4. Conclusion and Future Perspectives

There is still very much that is incompletely understood regarding Hsps in the eye, and this review has attempted to summarize the distribution of these proteins in the eye and there possible role in ocular pathogenesis. 

It has been suggested that upregulation and phosphorylation of Hsp70 can help in wound healing of the cornea after UV damage [21]. It has been shown that if the cells are preconditioned with arsenite, a Hsp27 agonist, prior to UVB exposure the level of cell death is reduced [21]. Kim et al. [4] found that introducing heat shock six hours before a photo refractive keratectomy procedure would increase the amount of Hsp70 and thereby minimize the loss of corneal fibroblast due to the prevention of cell apoptosis and necrosis. It was found that the healing of the corneal wound was accomplished without any problems, suggesting it might be an effective preoperative treatment prior to surgery. They also proposed that this technique could be used to treat inflammatory diseases of the cornea [4]. 

The decreased transparency in the lens with age seems to be due to the loss of alpha-crystallin function as a protective heat shock protein. Therefore, if prolonged function of alpha-crystallin can be stimulated, a delay of the age-related cataract might be possible. One way to manage this is by using proteasome inhibitors [90]. However, the actual association between cataract development and Hsps has to be more fully understood before it is possible to determine a preventative or treatment strategy that can be effectively applied to patients. 

On the perspective that the Hsp27 level is increased in the glaucomatous optic nerve head, Yu et al. [91] suggested inhibition of factors such as H_2_O_2_ and TGF-beta2 that promote Hsp27 may be beneficial in glaucoma treatment. However, before it can be fully understood how regulation of Hsps can be beneficial in preventing and treating glaucoma, the pathology of this disease need to be further clarified. 

The situation may be different with cancer since here loss of normal apoptosis seems to be one of the chief causes of the disease. Therefore, the compounds that can down- or upregulate Hsp levels and the heat shock response might be useful. It seems that there are currently two main strategies for cancer treatment with regard to the Hsps. One is modification of the Hsp expression and the other is to use Hsp to present the tumor antigen to the body's immune system [80]. However, since Hsps are not only associated with causing cancer, the best treatment would be the one that targets the cells with high Hsp levels that correspond with the cancer cells. The drug Quercetin, that downregulates Hsp70, has shown this ability in pancreatic cancer by selectively decreasing the cancer cells viability without affecting the normal ductal cells, since it did not affect the basal ductal cell Hsp70 level [92]. Further investigation of this finding and fundamental Hsp function with respect to ocular tumors is necessary.

## Figures and Tables

**Table 1:** Overview of heat shock protein ocular location and involvement in diseases of the eye.

	Hsp subtype	Diseases
Cornea	Hsp27 Hsp60 Hsp70 Hsp72 Hsp90	(i) Epithelial wound healing: Hsp70 has been suggested to be involved in wound healing of the cornea. (ii) UV radiation: the level of Hsp27 is increased in the corneal epithelium following UVB radiation and is suggested to play a protective role against such damaging radiation.

Lens	Hsp27 Alpha-crystallin Hsp40 Hsp70	(i) Cataract: Hsps have been suggested to play a significant role in cataract development most likely by either mutation of alpha-crystallin or by alpha-crystallin degeneration with age.

Retina	Hsp27 Hsp70 Hsp40 Hsp84 Hsp86 Hsp90	(i) Age-related macular degeneration (AMD): Hsp27 and alpha-crystallin are suggested to play a role in AMD after having been found to be induced and elevated in retina samples affected by the disease. (ii) Diabetic retinopathy. (iii) Glaucoma, light injury: possibly neuroprotective.

Choroid	Hsp-Beta1	

Other		(i) Ocular cancer: various ocular cancers are associated with increased levels of Hsps. It is not clear whether Hsps play a role in cancer development or are increased as a result of secondary homeostatic mechanisms. (a) Retinoblastoma: high expression of alpha-crystallin, Hsp70, and -90 have been found. (b) Uveal melanoma: increased levels of Hsp-beta1, -60, -70, and -90 have been detected. Hsp27 has been found increased, and low or not detectable.
		(ii) Glaucoma: Hsp27, -60, -70, and -72 have been found increased in glaucomatous eyes and pressure induced ischemic rodent eyes. However, it is unclear if Hsps confer cellular protection.

## References

[1] Hartl FU, Hayer-Hartl M (2002). Molecular chaperones in the cytosol: from nascent chain to folded protein. *Science*.

[2] Han MJ, Yun H, Lee SY (2008). Microbial small heat shock proteins and their use in biotechnology. *Biotechnology Advances*.

[3] Groenen PJTA, Merck KB, De Jong WW, Bloemendal H (1994). Structure and modifications of the junior chaperone *α*-crystallin—from lens transparency to molecular pathology. *European Journal of Biochemistry*.

[4] Kim YS, Han JA, Cheong TB, Ryu JC, Kim JC (2004). Protective effect of heat shock protein 70 against oxidative stresses in human corneal fibroblasts. *Journal of Korean Medical Science*.

[5] Lindquist S, Craig EA (1988). The heat-shock proteins. *Annual Review of Genetics*.

[6] Georgopoulos C, Welch WJ (1993). Role of the major heat shock proteins as molecular chaperones. *Annual Review of Cell Biology*.

[7] Li Y, Roth S, Laser M, Ma JX, Crosson CE (2003). Retinal preconditioning and the induction of heat-shock protein 27. *Investigative Ophthalmology and Visual Science*.

[8] Dasgupta S, Hohman TC, Carper D (1992). Hypertonic stress induces *α*B-crystallin expression. *Experimental Eye Research*.

[9] Omar R, Pappolla M (1993). Oxygen free radicals as inducers of heat shock protein synthesis in cultured human neuroblastoma cells: relevance to neurodegenerative disease. *European Archives of Psychiatry and Clinical Neuroscience*.

[10] Morimoto RI (1998). Regulation of the heat shock transcriptional response: cross talk between a family of heat shock factors, molecular chaperones, and negative regulators. *Genes and Development*.

[11] Wu C (1995). Heat shock transcription factors: structure and regulation. *Annual Review of Cell and Developmental Biology*.

[12] Gotoh T, Terada K, Oyadomari S, Mori M (2004). hsp70-DnaJ chaperone pair prevents nitric oxide- and CHOP-induced apoptosis by inhibiting translocation of Bax to mitochondria. *Cell Death and Differentiation*.

[13] Beere HM, Wolf BB, Cain K (2000). Heat-shock protein 70 inhibits apoptosis by preventing recruitment of procaspase-9 to the Apaf-1 apoptosome. *Nature Cell Biology*.

[14] Hsu AOL, Murphy CT, Kenyon C (2003). Regulation of aging and age-related disease by DAF-16 and heat-shock factor. *Science*.

[15] Jäättelä M (1999). Heat shock proteins as cellular lifeguards. *Annals of Medicine*.

[16] Gain P, Thuret G, Chiquet C, Dumollard JM, Mosnier JF, Campos L (2001). In situ immunohistochemical study of Bcl-2 and heat shock proteins in human corneal endothelial cells during corneal storage. *British Journal of Ophthalmology*.

[17] Young JC (2010). Mechanisms of the Hsp70 chaperone system. *Biochemistry and Cell Biology*.

[18] Dang W, Hu YH, Zhang M, Sun LI (2010). Identification and molecular analysis of a stress-inducible Hsp70 from Sciaenops ocellatus. *Fish and Shellfish Immunology*.

[19] Ishihara M, Sato M, Sato S, Arai T, Obara M, Kikuchi M (2004). Assessment of expressions of heat shock protein (HSP 72) and apoptosis after ArF excimer laser ablation of the cornea. *Journal of Biomedical Optics*.

[20] Shi B, Isseroff RR (2006). Arsenite pre-conditioning reduces UVB-induced apoptosis in corneal epithelial cells through the anti-apoptotic activity of 27 kDa heat shock protein (HSP27). *Journal of Cellular Physiology*.

[21] Shi B, Han B, Schwab IR, Isseroff RR (2006). UVB irradiation-induced changes in the 27-kd heat shock protein (HSP27) in human corneal epithelial cells. *Cornea*.

[22] Ko MK, Kay EP (1999). Hsp47-dependent and -independent intracellular trafficking of type I collagen in corneal endothelial cells. *Molecular Vision*.

[23] Gu X, Ko MK, Kay EP (1999). Intracellular interaction of Hsp47 and type I collagen in corneal endothelial cells. *Investigative Ophthalmology and Visual Science*.

[24] Nakai A, Satoh M, Hirayoshi K, Nagata K (1992). Involvement of the stress protein HSP47 in procollagen processing in the endoplasmic reticulum. *Journal of Cell Biology*.

[25] Nagata K (1996). Hsp47: a collagen-specific molecular chaperone. *Trends in Biochemical Sciences*.

[26] Kasagi Y, Yamashita H (2002). HSP47 expression in cornea after excimer laser photoablation. *Japanese Journal of Ophthalmology*.

[27] Krishnan K, Kathiresan T, Raman R (2007). Ubiquitous lens *α*-, *β*-, and *γ*-crystallins accumulate in anuran cornea as corneal crystallins. *Journal of Biological Chemistry*.

[28] Bagchi M, Katar M, Maisel H (2001). Heat shock proteins of adult and embryonic human ocular lenses. *Journal of Cellular Biochemistry*.

[29] Chiu K, Zhou Y, Yeung SC (2010). Up-regulation of crystallins is involved in the neuroprotective effect of wolfberry on survival of retinal ganglion cells in rat ocular hypertension model. *Journal of Cellular Biochemistry*.

[30] Rothstein H, Bagchi M (1969). Synthesis of macromolecules in epithelial cells of the cultured amphibian lens. 3. Involvement of protein synthesis in mitotic activation. *Archives Internationales de Physiologie et de Biochimie*.

[31] Wistow GJ, Piatigorsky J (1988). Lens crystallins: the evolution and expression of proteins for a highly specialized tissue. *Annual Review of Biochemistry*.

[32] Horwitz J (1993). The function of alpha-crystallin. *Investigative Ophthalmology and Visual Science*.

[33] Maiti M, Kono M, Chakrabarti B (1988). Heat-induced changes in the conformation of *α*- and *β*-crystallins: unique thermal stability of *α*-crystallin. *FEBS Letters*.

[34] Heys KR, Friedrich MG, Truscott RJW (2007). Presbyopia and heat: changes associated with aging of the human lens suggest a functional role for the small heat shock protein, *α*-crystallin, in maintaining lens flexibility. *Aging Cell*.

[35] Wistow G (1985). Domain structure and evolution in *α*-crystallins and small heat-shock proteins. *FEBS Letters*.

[36] Laganowsky A, Benesch JLP, Landau M (2010). Crystal structures of truncated alphaA and alphaB-crystallins reveal structural mechanisms of polydispersity important for eye lens function. *Protein Science*.

[37] Raman B, Ramakrishna T, Rao CM (1995). Temperature dependent chaperone-like activity of alpha-crystallin. *FEBS Letters*.

[38] Jakob U, Gaestel M, Engel K, Buchner J (1993). Small heat shock proteins are molecular chaperones. *Journal of Biological Chemistry*.

[39] Baneyx F, Mujacic M (2004). Recombinant protein folding and misfolding in Escherichia coli. *Nature Biotechnology*.

[40] Ehrnsperger M, Gräber S, Gaestel M, Buchner J (1997). Binding of non-native protein to Hsp25 during heat shock creates a reservoir of folding intermediates for reactivation. *EMBO Journal*.

[41] Lee GJ, Roseman AM, Saibil HR, Vierling E (1997). A small heat shock protein stably binds heat-denatured model substrates and can maintain a substrate in a folding-competent state. *EMBO Journal*.

[42] Kim JH, Yu YS, Chung H, Heo JW, Seo JS (2003). Effect of the absence of heat shock protein 70.1 (hsp70.1) on retinal photic injury. *Korean Journal of Ophthalmology*.

[43] Kojima M, Hoshimaru M, Aoki T (1996). Expression of heat shock proteins in the developing rat retina. *Neuroscience Letters*.

[44] Tytell M, Barbe MF, Brown IR (1994). Induction of heat shock (stress) protein 70 and its mRNA in the normal and light-damaged rat retina after whole body hyperthermia. *Journal of Neuroscience Research*.

[45] Tezel G, Wax MB (2000). The mechanisms of hsp27 antibody-mediated apoptosis in retinal neuronal cells. *Journal of Neuroscience*.

[46] Tanaka Y, Kobayashi K, Kita M, Kinoshita S, Imanishi J (1995). Messenger RNA expression of heat shock proteins (HSPs) during ocular development. *Current Eye Research*.

[47] Dinh HK, Zhao B, Schuschereba ST, Merrill G, Bowman PD (2001). Gene expression profiling of the response to thermal injury in human cells. *Physiol Genomics*.

[48] Ochoa GH, Clark YM, Matsumoto B, Torres-Ruiz JA, Robles LJ (2002). Heat shock protein 70 and heat shock protein 90 expression in light- and dark-adapted adult octopus retinas. *Journal of Neurocytology*.

[49] Kim JH, Kim JH, Yu YS, Jeong SM, Kim KW (2005). Protective effect of heat shock proteins 70.1 and 70.3 on retinal photic injury after systemic hyperthermia. *Korean Journal of Ophthalmology*.

[50] Zamora DO, Riviere M, Choi D (2007). Proteomic profiling of human retinal and choroidal endothelial cells reveals molecular heterogeneity related to tissue of origin. *Molecular Vision*.

[51] Ko JA, Yanai R, Quan WY, Morishige N, Nishida T (2008). Up-regulation of HSP70 by the fibronectin-derived peptide PHSRN in human corneal epithelial cells. *Biochemical and Biophysical Research Communications*.

[52] Oberringer M, Baum HP, Jung V (1995). Differential expression of heat shock protein 70 in well healing and chronic human wound tissue. *Biochemical and Biophysical Research Communications*.

[53] Mushtaq S, Naqvi ZA, Siddiqui AA, Palmberg C, Shafqat J, Ahmed N (2007). Changes in albumin precursor and heat shock protein 70 expression and their potential role in response to corneal epithelial wound repair. *Proteomics*.

[54] Mushtaq S, Naqvi ZA, Siddiqui AA, Ahmed N (2011). Albumin precursor and Hsp70 modulate corneal wound healing in an organ culture model. *Acta Histochemica*.

[55] Schein OD, Vicencio C, Munoz B (1995). Ocular and dermatologic health effects of ultraviolet radiation exposure from the ozone hole in Southern Chile. *American Journal of Public Health*.

[56] Kulms D, Zeise E, Pöppelmann B, Schwarz T (2002). DNA damage, death receptor activation and reactive oxygen species contribute to ultraviolet radiation-induced apoptosis in an essential and independent way. *Oncogene*.

[57] Ringvold A (1998). Corneal epithelium and UV-protection of the eye. *Acta Ophthalmologica Scandinavica*.

[58] Shimmura S, Suematsu M, Shimoyama M, Tsubota K, Oguchi Y, Ishimura Y (1996). Subthreshold UV radiation-induced peroxide formation in cultured corneal epithelial cells: the protective effects of lactoferrin. *Experimental Eye Research*.

[59] Kennedy M, Kim KH, Harten B (1997). Ultraviolet irradiation induces the production of multiple cytokines by human corneal cells. *Investigative Ophthalmology and Visual Science*.

[60] Francis PJ, Berry V, Moore AT, Bhattacharya S (1999). Lens biology: development and human cataractogenesis. *Trends in Genetics*.

[61] Andley UP (2009). Effects of *α*-crystallin on lens cell function and cataract pathology. *Current Molecular Medicine*.

[62] Bhat SP (2003). Crystallins, genes and cataract. *Progress in Drug Research*.

[63] Derham BK, Harding JJ (1999). *α*-crystallin as a molecular chaperone. *Progress in Retinal and Eye Research*.

[64] Ishikura R, Kato S, Nagata M, Tamai A, Ohama E (1999). Expression of stress-response protein 60 in lens epithelial cells in atopic cataract. *Japanese Journal of Ophthalmology*.

[65] Lohse AW, Dienes HP, Herkel J, Hermann E, Van Eden W, Zum Buschenfelde KHM (1993). Expression of the 60 kDa heat shock protein in normal and inflamed liver. *Journal of Hepatology*.

[66] Fagerholm P, Palmquist BM, Philipson B (1984). Atopic cataract: changes in the lens epithelium and subcapsular cortex. *Graefe’s Archive for Clinical and Experimental Ophthalmology*.

[67] Kaarniranta K, Salminen A, Eskelinen EL, Kopitz J (2009). Heat shock proteins as gatekeepers of proteolytic pathways-Implications for age-related macular degeneration (AMD). *Ageing Research Reviews*.

[68] Strunnikova N, Baffi J, Gonzalez A, Silk W, Cousins SW, Csaky KG (2001). Regulated heat shock protein 27 expression in human retinal pigment epithelium. *Investigative Ophthalmology and Visual Science*.

[69] Nakata K, Ohji M, Ikuno Y (2004). Wide-angle viewing lens for vitrectomy. *American Journal of Ophthalmology*.

[70] Pineda R, Chan CC, Ni M (1993). Human retinoblastoma cells express *α*B-crystallin in vivo and in vitro. *Current Eye Research*.

[71] Kase S, Parikh JG, Rao N (2009). Expression of *α*-crystallin in retinoblastoma. *Archives of Ophthalmology*.

[72] Jiang LB, Liu XQ, Li B (2008). Heat shock proteins and survivin: relationship and effects on proliferation index of retinoblastoma cells. *Histology and Histopathology*.

[73] Chen CF, Chen Y, Dai K, Chen PL, Riley DJ, Lee WH (1996). A new member of the hsp90 family of molecular chaperones interacts with the retinoblastoma protein during mitosis and after heat shock. *Molecular and Cellular Biology*.

[74] Pardo M, García A, Thomas B (2005). Proteome analysis of a human uveal melanoma primary cell culture by 2-DE and MS. *Proteomics*.

[75] Zuidervaart W, Hensbergen PJ, Wong MC (2006). Proteomic analysis of uveal melanoma reveals novel potential markers involved in tumor progression. *Investigative Ophthalmology and Visual Science*.

[76] Missotten GS, Journée-de Korver JG, De Wolff-Rouendaal D, Keunen JE, Schlingemann RO, Jager MJ (2003). Heat shock protein expression in the eye and in uveal melanoma. *Investigative Ophthalmology and Visual Science*.

[77] Coupland SE, Vorum H, Mandal N (2010). Proteomics of uveal melanomas suggests HSP-27 as a possible surrogate marker of chromosome 3 loss. *Investigative Ophthalmology & Visual Science*.

[78] Whitesell L, Lindquist SL (2005). HSP90 and the chaperoning of cancer. *Nature Reviews Cancer*.

[79] Soo ETL, Yip GWC, Lwin ZM, Kumar SD, Bay BH (2008). Heat shock proteins as novel therapeutic targets in cancer. *In Vivo*.

[80] Ciocca DR, Calderwood SK (2005). Heat shock proteins in cancer: diagnostic, prognostic, predictive, and treatment implications. *Cell Stress and Chaperones*.

[81] Hitchings RA, Spaeth GL (1977). Fluorescein angiography in chronic simple and low tension glaucoma. *British Journal of Ophthalmology*.

[82] Quigley HA, Nickells RW, Kerrigan LA, Pease ME, Thibault DJ, Zack DJ (1995). Retinal ganglion cell death in experimental glaucoma and after axotomy occurs by apoptosis. *Investigative Ophthalmology and Visual Science*.

[83] Garcia-Valenzuela E, Shareef S, Walsh J, Sharma SC (1995). Programmed cell death of retinal ganglion cells during experimental glaucoma. *Experimental Eye Research*.

[84] Tezel G, Hernandez MR, Wax MB (2000). Immunostaining of heat shock proteins in the retina and optic nerve head of normal and glaucomatous eyes. *Archives of Ophthalmology*.

[85] Windisch BK, LeVatte TL, Archibald ML, Chauhan BC (2009). Induction of heat shock proteins 27 and 72 in retinal ganglion cells after acute pressure-induced ischaemia. *Clinical and Experimental Ophthalmology*.

[86] Huang W, Fileta JB, Filippopoulos T, Ray A, Dobberfuhl A, Grosskreutz CL (2007). Hsp27 phosphorylation in experimental glaucoma. *Investigative Ophthalmology and Visual Science*.

[87] Qing G, Duan X, Jiang Y (2005). Heat shock protein 72 protects retinal ganglion cells in rat model of acute glaucoma. *Yan Ke Xue Bao*.

[88] Tezel G, Seigel GM, Wax MB (1998). Autoantibodies to small heat shock proteins in glaucoma. *Investigative Ophthalmology and Visual Science*.

[89] Wax MB, Tezel G, Kawase K, Kitazawa Y (2001). Serum autoantibodies to heat shock proteins in glaucoma patients from Japan and the United States. *Ophthalmology*.

[90] Awasthi N, Wagner BJ (2005). Upregulation of heat shock protein expression by proteasome inhibition: an antiapoptotic mechanism in the lens. *Investigative Ophthalmology and Visual Science*.

[91] Yu AL, Fuchshofer R, Birke M, Kampik A, Bloemendal H, Welge-Lüssen U (2008). Oxidative stress and TGF-*β*2 increase heat shock protein 27 expression in human optic nerve head astrocytes. *Investigative Ophthalmology and Visual Science*.

[92] Aghdassi A, Phillips P, Dudeja V (2007). Heat shock protein 70 increases tumorigenicity and inhibits apoptosis in pancreatic adenocarcinoma. *Cancer Research*.

